# 3D visualization technology for Learning human anatomy among medical students and residents: a meta- and regression analysis

**DOI:** 10.1186/s12909-024-05403-4

**Published:** 2024-04-26

**Authors:** Junming Wang, Wenjun Li, Aishe Dun, Ning Zhong, Zhen Ye

**Affiliations:** 1 Department of Health Management, The First Affiliated Hospital, Shandong Provincial Qianfoshan Hospital, Shandong First Medical University, 250013 Jinan, Shandong China; 2 School of clinical and basic medicine, Shandong First Medical University, Jinan, China; 3 School of Stomatology, Shandong First Medical University, Jinan, China

**Keywords:** 3D visualization technology, Human anatomy, Meta-analysis, Regression analysis, Medical education

## Abstract

**Background:**

3D visualization technology applies computers and other devices to create a realistic virtual world for individuals with various sensory experiences such as 3D vision, touch, and smell to gain a more effective understanding of the relationships between real spatial structures and organizations. The purpose of this study was to comprehensively evaluate the effectiveness of 3D visualization technology in human anatomy teaching/training and explore the potential factors that affect the training effects to better guide the teaching of classroom/laboratory anatomy.

**Methods:**

We conducted a meta-analysis of randomized controlled studies on teaching human anatomy using 3D visualization technology. We extensively searched three authoritative databases, PubMed, Web of Science, and Embase; the main outcomes were the participants’ test scores and satisfaction, while the secondary outcomes were time consumption and enjoyment. Heterogeneity by I² was statistically determined because I²> 50%; therefore, a random-effects model was employed, using data processing software such as RevMan, Stata, and VOSviewer to process data, apply standardized mean difference and 95% confidence interval, and subgroup analysis to evaluate test results, and then conduct research through sensitivity analysis and meta-regression analysis.

**Results:**

Thirty-nine randomized controlled trials (2,959 participants) were screened and included in this study. The system analysis of the main results showed that compared with other methods, including data from all regions 3D visualization technology moderately improved test scores as well as satisfaction and enjoyment; however, the time that students took to complete the test was not significantly reduced. Meta-regression analysis also showed that regional factorsaffected test scores, whereas other factors had no significant impact. When the literature from China was excluded, the satisfaction and happiness of the 3D virtual-reality group were statistically significant compared to those of the traditional group; however, the test results and time consumption were not statistically significant.

**Conclusion:**

3D visualization technology is an effective way to improve learners’ satisfaction with and enjoyment of human anatomical learning, but it cannot reduce the time required for testers to complete the test. 3D visualization technology may struggle to improve the testers’ scores. The literature test results from China are more prone to positive results and affected by regional bias.

**Supplementary Information:**

The online version contains supplementary material available at 10.1186/s12909-024-05403-4.

## Introduction

Human anatomy is a compulsory course for both clinical and medical students; it is important for clinicians—especially surgeons—to master the anatomy of the human body. However, inadequate anatomical knowledge among medical students and young residents has been reported [1]. This occurs for several reasons: limited teaching time in anatomy in undergraduate education, which is associated with increased costs; limited availability of cadavers; and reduced exposure to traditional autopsies [2]. Traditional learning of anatomy is based on elements such as cadaver specimen, regional/ topography anatomical models, and two-dimensional atlases. Two-dimensional atlases lack a sense of three-dimensional space, and it is difficult to reflect the real spatial structure and relationship among organizations. Autopsies offer a complete visual and tactile experience of anatomical learning that is essentially three-dimensional. The traditional cadaverspecimen has the following shortcomings: shortage of cadaver sources; irritant of antiseptic reagent; and nerves and blood vessels lacking a clear holistic view. Features such as stereo vision, dynamic exploration, and tactile feedback are essential for three-dimensional anatomy [3]. As patients are 3D objects, medical treatment and education involve learning and applying 3D information.

Therefore, digital 3D visualization technology using computer imaging has shown potential educational value, owing to its high fidelity to organizations. 3D visualization technologies include virtual reality (VR), augmented reality (AR), and mixed reality (MR), among others. VR is a process of visualizing a computer-generated environment in an interactive manner using software and hardware [4].AR is an experience that involves superimposition of digital elements such as graphics, audio, and other sensory enhancements onto video streams of the real world with real-time interaction between the user and the digital elements. Although VR replaces the real-world environment with a virtual world, AR supplements a user’s perception of the real world in an immersive manner without obscuring it completely [5].MR is a hybrid of the real and virtual worlds. MR is created when computer processing combines the user’s inputs and environment to create an immersive environment in which physical and virtual objects coexist and interact in real time [6].An example of this technology is the superposition of information or 3D models onto a head-mounted display (HMD); however, MR HMDs do not obfuscate the real world [5].

The AR, VR, and MR concepts can be distinguished based on three criteria: immersion, interaction, and information [7]. Immersion refers to the nature of the user experience brought by the technology. Although VR provides an entirely immersive virtual experience, AR augments a real-world view using virtual information. MR performs spatial mapping between the real and virtual worlds in real time. Interaction refers to the types of interactions that are feasible through the use of technology. VR allows interactions with virtual objects, whereas AR enables interactions with physical objects. MR allows interactions with both physical and virtual objects. Information refers to the type of data handled during visualization. In VR, a displayed virtual object is registered in a virtual 3D space. AR provides virtual annotations in real time within a user’s environment. In MR, the displayed virtual object is registered in 3D space and time, with a correlation to the user’s environment in the real world [5].

In contrast to 2D imaging methods, such as textbook diagrams, photographs, digital CT, and MRI scans, the most obvious advantage of 3D visualization is its ability to view the spatial relationships between different anatomical structures from numerous viewpoints and angles. While diagrams and radiological imaging provide static snapshots, videos using 3D visualization offer the possibility of a narrative timeline where students can pause, rewind, or fast-forward at their convenience. In addition, through 3D image reconstruction, the two-dimensional information contained in images generated by CT, MRI, X-ray, etc. can be converted into three-dimensional information, thereby helping doctors restore the three-dimensional shape of various tissues [8].3D images alsooffer the ability to rotate, flip, and invert a viewed structure [9].

To better grasp anatomical knowledge, physical3D printing technology was introduced in our previous two studies [10, 11], and it can be used as a good auxiliary tool to learn anatomy structure. In our previous meta-analysis, for achievement tests, we found no statistical difference between the 3D printing model group and the 3D visualization group [10].

Despite the surge in the use of digital 3D visualization technology with computer imaging in medical anatomy education, a comprehensive evaluation of its effectiveness through randomized trials is lacking [12]. To better understand the effectiveness of digital 3D visualization technology using computer imaging in anatomy teaching, we systematically evaluated published literature to better guide anatomy teaching.

Given the above considerations, this study aims to

1. Provide a comprehensive summary of research evaluating the educational effectiveness (test scores and time consumption) of 3D visualization technology in medical anatomy education compared to conventional teaching methods.
2. Provide a comprehensive summary of research evaluating the popularity (satisfaction and enjoyment) of 3D visualization technology in medical anatomy education compared to conventional teaching methods.
3. Explore the potential factors affecting the effect of 3D visualization application.
4. There have been many studies on 3D visualization technology, but their conclusions and results differ. Our study differs from previous studies, and we have proposed new goals and perspectives and hope to provide a reference for future research on this technology.

## Materials and methods

This meta-analysis complies with the Preferred Reporting Items for Systematic Reviews and Meta-Analyses (PRISMA) guidelines [13].

### Literature search

PubMed, Web of Science, and EBSCO (with subset databases including MEDLINE Ultimate, MEDLINE, Academic Search Premier, APA Psyclnfo, and ERIC) were searched for relevant literature. The search keywords were as follows: (“3D” or “Three-dimensional”) and (“visualization” or “visuospatial” or “stereoscop” or “stereocept” or “stereopsis” or “stereoscopic vision” or “virtual reality”) and (“medical” or “medicine”) and(“education” or “teaching”) and (“students” or “residents”) and (“group” or “study”) and (“anatomy” or “dissect”). The language setting for the literature searched was English. The deadline for publication of the included studies was July 2023.

### Literature screening and data extraction

We set the inclusion criteria for the literature to be included as follows: (1) types of research: randomized controlled trials; (2) research objects: medical students or residents; (3) intervention measures: in the experimental group, 3D imaging was used to display the anatomical structure; (4) outcome index: test scores, satisfaction, time consumption, enjoyment; (5) research articles on the teaching or training of human anatomy; (6) the population receiving training included medical students or resident physicians; and (7) a control group was required. Exclusion criteria were as follows: (1) experiments lacking comparative studies; (2) research including animal anatomy; and (3) reviews, case reports, and studies for which valid data could not be extracted.

We conducted preliminary and fine screening of the retrieved literature. Preliminary screening involved reading titles and abstracts and removing the literature that clearly did not meet the requirements. In the next step, we read the full text, and if we encountered difficulties, we negotiated with another participant to solve them together.

### Quality assessment of the included literature

For the quality assessment of the included literature, we used Review Manager 5.3 (https://www.duote.com/soft/911598.html) to evaluate the risk level after reading the full articles.

The assessment methods included the following indicators: (1) random sequence generation, (2) allocation concealment, (3) blinding of participants and personnel, (4) blinding of outcome assessment, (5) incomplete outcome data, (6) selective reporting, and (7) other bias. Each indicator was evaluated using the following three options: high, low, or unclear risk. If disagreement arose, it was resolved through negotiation; therefore, the lower the risk, the higher the quality of the literature.

### Text mining of the included literature

We used VOSviewer 1.6.19 (https://www.vosviewer.com/download) to mine the time, country, and anatomical parts of the included literature.VOSviewer can be used to construct maps of authors or journals based on co-citation data or maps of keywords based on co-occurrence data [14].

**Fig. 1:**
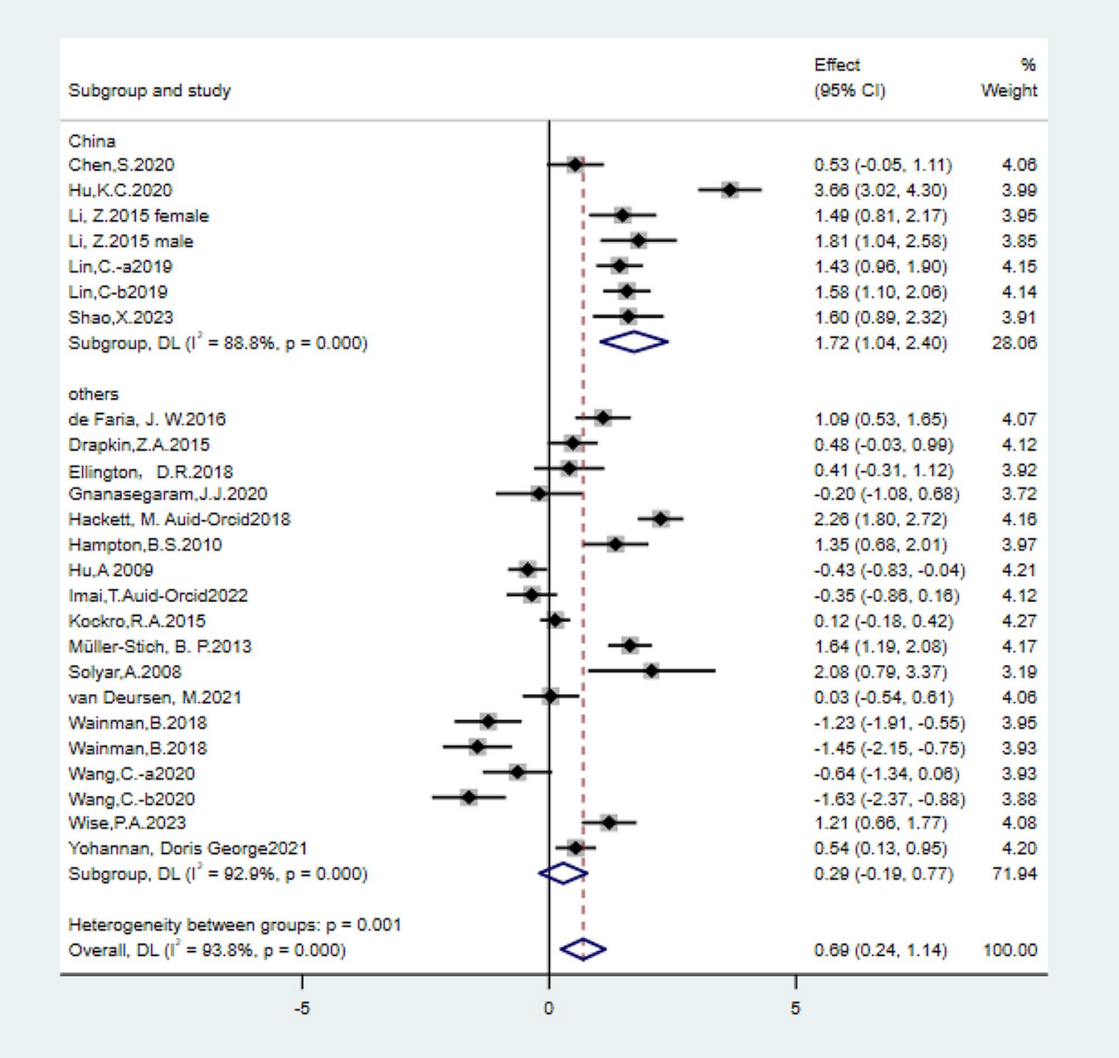
Regional distribution of literature sources

In this study, VOSviewer displays maps in two ways: scatter and density views. In the scatter view, items are indicated by small circles; the more important the project, the larger the circle. If colors have been assigned to the items, each item’s circle is displayed in the item’s color [14]. For example, in Supplementary Figure S1, each circle represents the country to which the randomized controlled trial belongs, and the larger the circle, the more the literature from that country included in the study. In the density view, the approach is similar to that in the scatter view. For example, in Fig. 1, each fluorescent circle represents an anatomical part corresponding to the randomized controlled trial, and the brighter the fluorescent color, the more documents on this anatomical part are included in this study.

### Combined analysis of data

In forest plots, the size of heterogeneity is described by the square of I. According to experience, heterogeneity is sometimes described as low when the square of I is less than 50%, medium when the square of I is 50–75%, and high when the square of I is more than 75% [15].

Egger’s regression is a tool used to detect research bias in meta-analysis. It can be used to test the bias of pleiotropic effect, and its slope coefficient provides an estimate of the causal effect [16].

The choice of meta-analysis model depends on the existence or non-existence of heterogeneity. With no heterogeneity (heterogeneity *p* < 0.10), a fixed-effects model is used; however, with heterogeneity (heterogeneity *p* < 0.10) in the study, the random-effects model should be used for meta-analysis [17].

A funnel diagram, the most common method for identifying publication bias, is a scatter diagram made of sample content (or reciprocal of the standard error of effect quantity) and effect quantity (or logarithm of effect quantity). The funnel graph asymmetry test evaluates specific types of heterogeneity and is more powerful in this case [18].

Meta-regression is a regression analysis of the effect value at the research level. It is used to identify and screen for heterogeneity, analyze its source, and provide a basis for subsequent subgroup analysis. The application condition of meta-regression analysis is not less than 10 studies, and in this study, we included 25. The region was divided into China and other countries, and time was divided into before 2018 and after 2018 (including 2018).

### Statistical analysis

In the forest map, we used the square of I to describe the heterogeneity of the data, and Egger’s test. For continuous data, due to different scoring standards, we used the standardized mean difference (SMD) to compare the results. For the joint analysis of continuous variables, we analyzed the averages (X) and standard deviations of the experimental and control groups. In addition, the statistical method used in meta-regression analysis is the t-test, which can test whether the predicted variables are significant. According to the size of heterogeneity, a random-effects model is used to merge the data. Finally, the stability of the data is evaluated by sensitivity analysis, in which the random-effects model is used; the statistical effect quantity is the OR value, and the 95% confidence interval (CI) statistical method is used. In case of no special explanation, a p-value of less than 0.05 is considered statistically significant.

## Results

### Literature screening

**Fig. 2:**
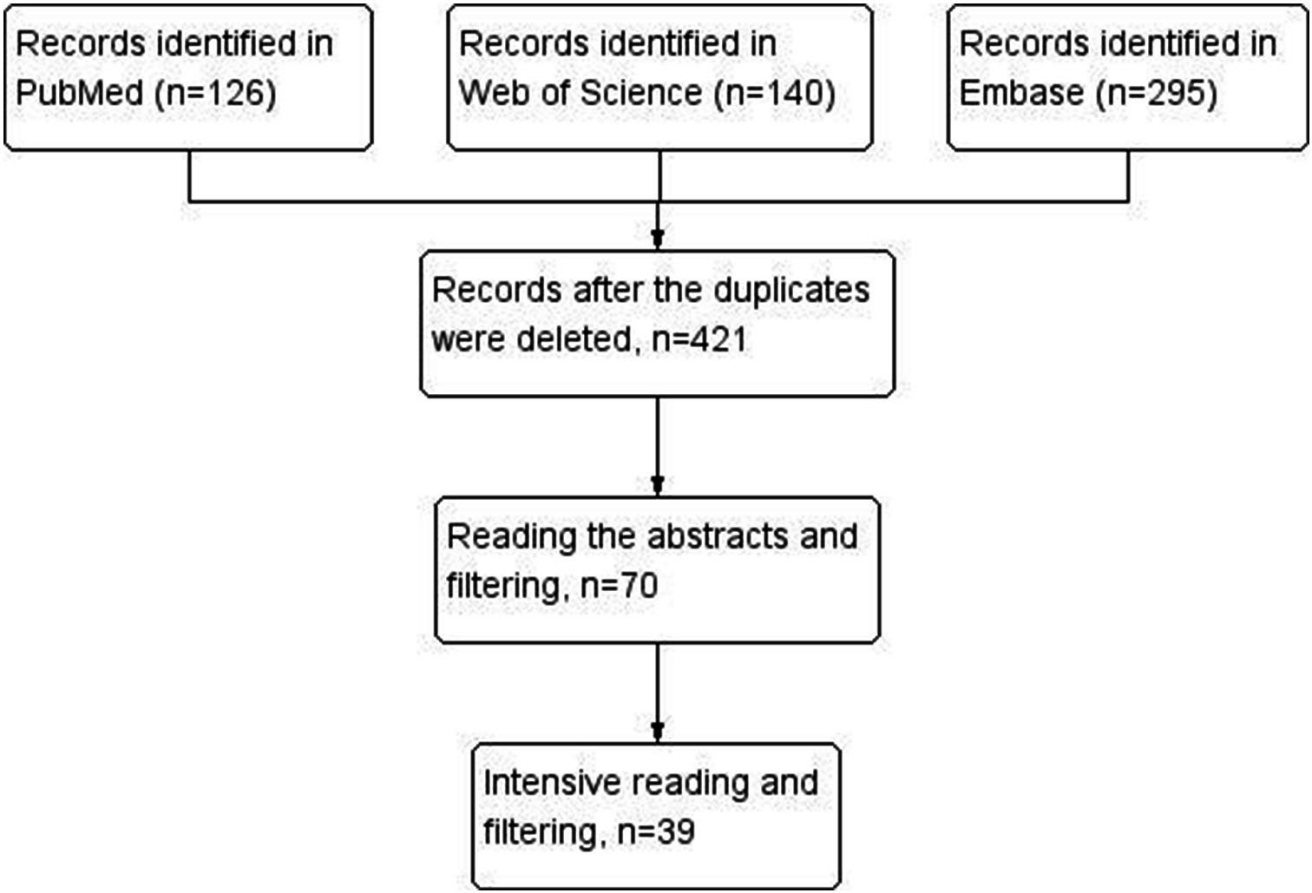
Flowchart of the search strategy

We downloaded the retrieved literature catalog as a whole, incorporated it into Endnote, summarized and merged it, and then divided it into two stages of literature screening, namely coarse screening and fine screening. By reading the articles’ abstracts, we could roughly screen them, and by downloading the full text for reading, we could refine the screening process. Based on the set search conditions, we retrieved 126 studies from the PubMed database, 140 studies from the Web of Science database, and 295 studies from other databases. After reading the titles and eliminating repetitive references, 39 articles remained (Fig. 2). Overall, 39 studies (Supplementary Table S1) met the inclusion requirements [1, 19–56]. These were 39 randomized controlled studies with 2,959 participants: eight were from the United States; seven from China; six from Canada; five from Germany; three from the Netherlands; two from France; and one each from Russia, Belgium, India, Switzerland, New Zealand, Tunisia, Turkey, Thailand, and Japan.

### Literature quality analysis

**Fig. 3:**
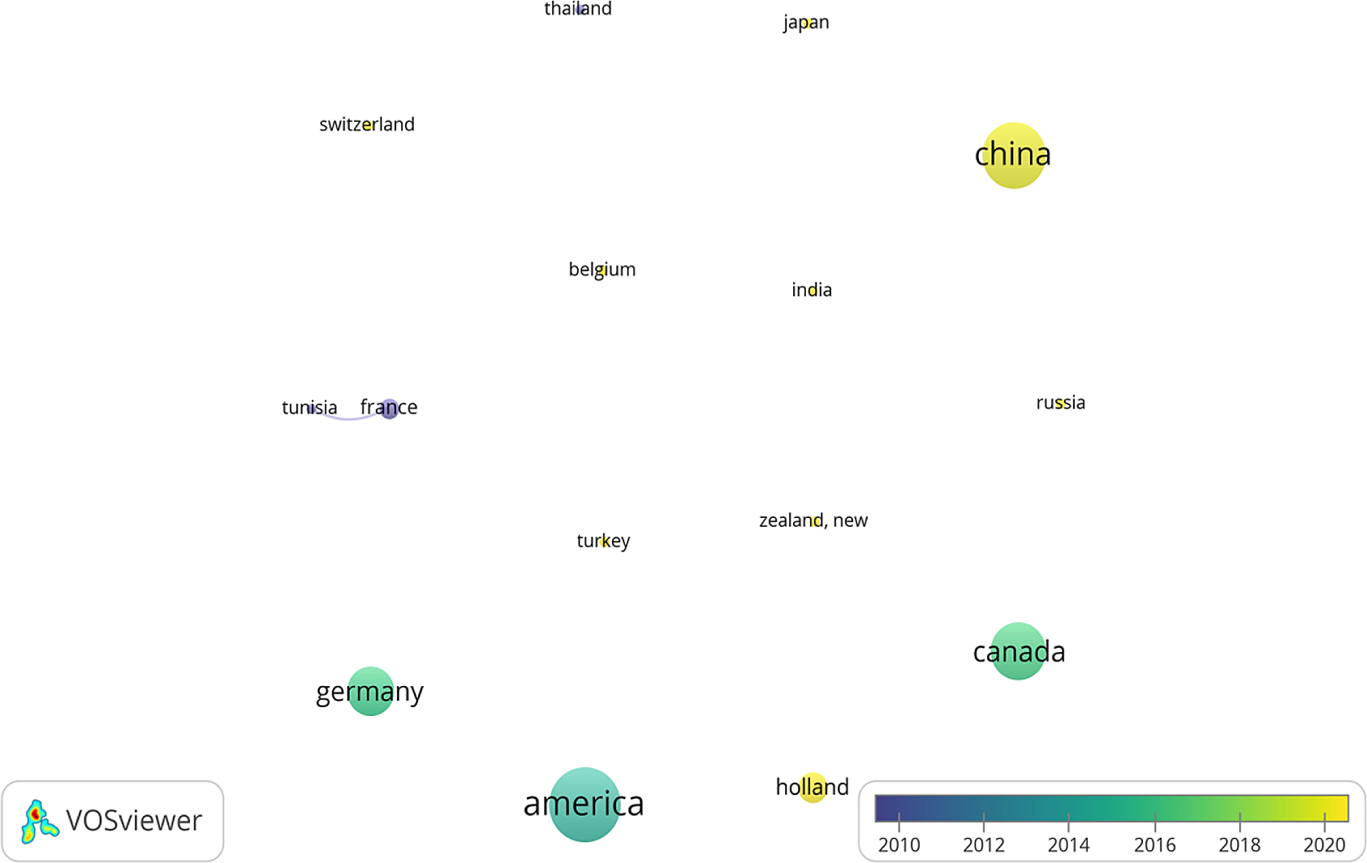
Risk of bias summary of included studies

As shown in the quality analysis, the risk of bias is relatively low in most studies (Fig. 3& Supplementary Figure S2). A few studies lack information on performance and detection bias [21, 23, 26, 38] as, due to the nature of the intervention, it was impractical to conduct blind checks on students and residents during the research process (selection bias). Most studies were determined to have a low risk of selection and low risk of attrition bias due to the complete data of the research results and the use of random selection for grouping [1, 19–23, 25–30, 34–37, 39–51]. The judgment of whether the research has selective reporting is based on whether the results are fully mentioned in the manuscript or discussion section. Three studies were judged to be at high risk of other bias as the experimental or control group had fewer than 10 participants [20, 24, 52]. Finally, one study was judged to have a high risk of performance bias due to the participants’ biased understanding of the assigned interventions during the study period [24].

### Literature information analysis

As shown in the Fig. 1, the United States, China, Canada, and Germany conducted more randomized controlled trials on this topic, followed by the Netherlands.

As shown in Supplementary Figure S1, a greater number of randomized controlled trial on this topic were conducted in the area of neuroanatomy, followed by head and neck, liver, and cardiac anatomy.

### Data merging of test scores

Based on StataMP 17 (64-bit), we made a score forest map, and all the studies reported the influence of intervention on test scores (of the 39 articles we cited, 35 included the influence on test scores, and the data of 25 articles were included).

**Fig. 4:**
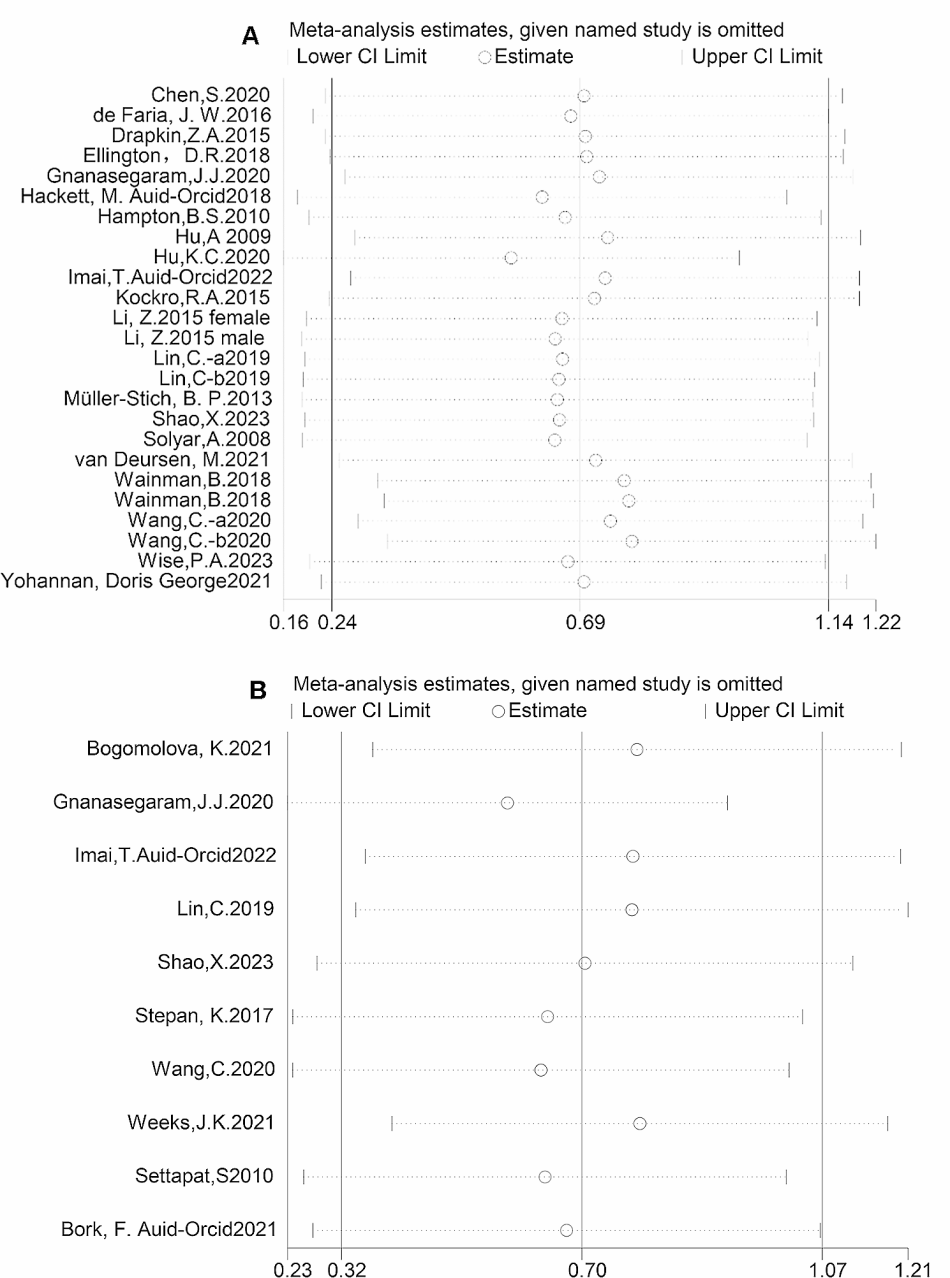
Comparison of the experimental and control groups for test scores

With regard to overall data consolidation, in the random-effects model, compared with traditional learning, 3D technology significantly improved learners’ test scores (SMD = 0.69, 95% CI = 0.24–1.14, *p* < 0.05, I^2^ = 93.8%, Fig. 4).

As subgroup analysis displayed, merging the literature data of China has statistical significance (SMD = 1.72, 95% CI = 1.04–2.40, *p* < 0.05, I^2^ = 88.8%, Fig. 4); however, merging data from regions outside of China shows no statistical significance (SMD = 0.29, 95% CI = -0.19–0.77, *p* < 0.05, I^2^ = 92.9%, Fig. 4).

The medical students subgroup displayed statistical significance (SMD = 0.68, 95% CI = -0.17–1.19, *p* < 0.05, I^2^ = 94%), while the residents subgroup showed no statistical significance (SMD = 0.74, 95% CI = -0.31–1.80, *p* < 0.05, I^2^ = 94.5%, Supplementary Figure S3).

### Data merging of satisfaction degree

**Fig. 5:**
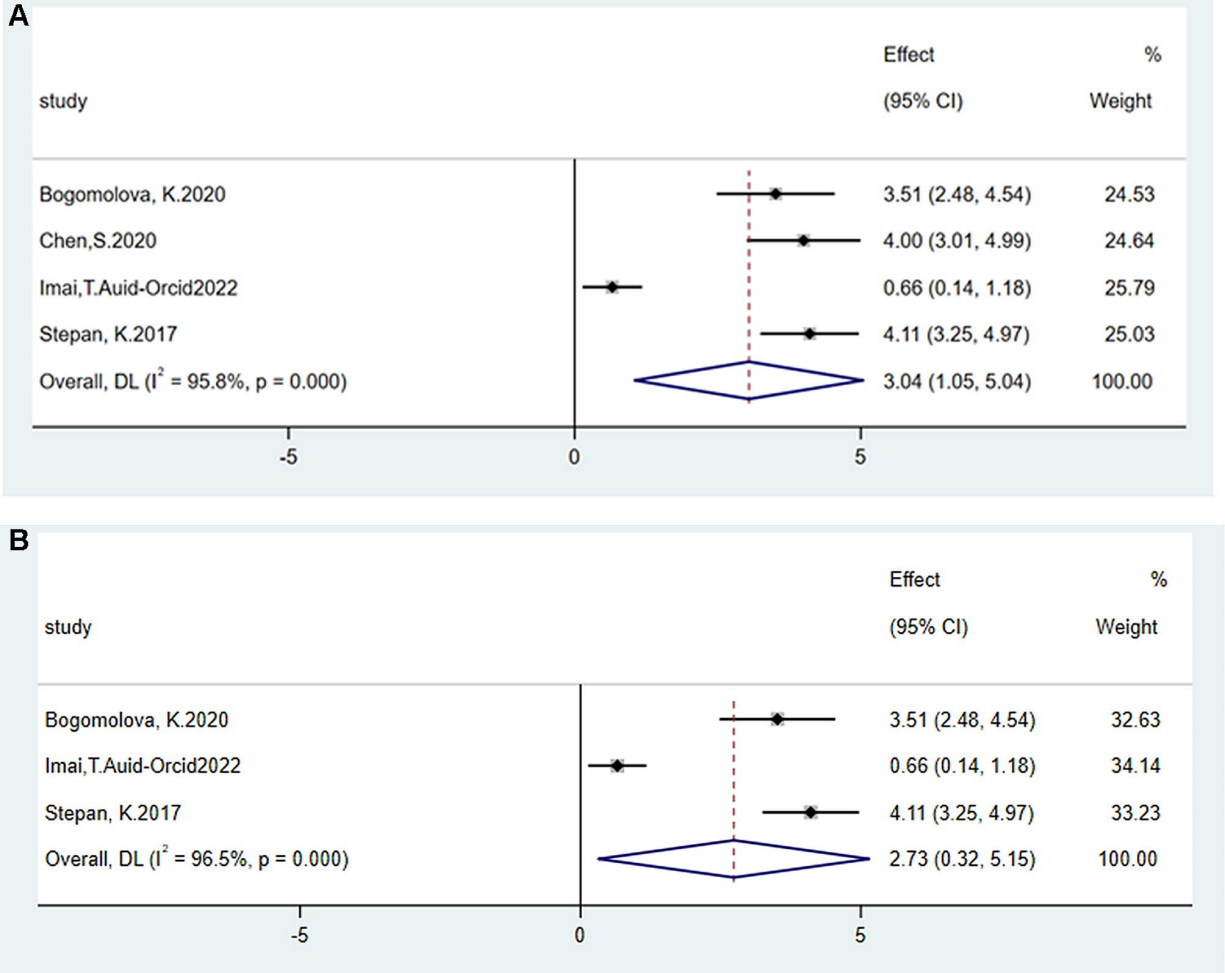
Comparison of the experimental and control groups for satisfaction outcome

Ten studies [24, 32, 36, 41, 43, 45, 48, 50, 52, 53] evaluated satisfaction as a secondary outcome (Fig. 5A). The summary results based on the random-effects model show that most students are more interested in learning through 3D methods than traditional or 2D teaching methods (SMD = 0.70, 95% CI = 0.32–1.07, *p* < 0.05, I^2^ = 69.0%), which may be related to the more intuitive experience given by 3D technology. If the literature from China is excluded, the 3D group has statistical significance as well (SMD = 0.79, 95% CI = 0.30–1.29, *p* < 0.05, I^2^ = 69.0%,Fig. 5B).

### Data merging of time and enjoyment degree

**Fig. 6:**
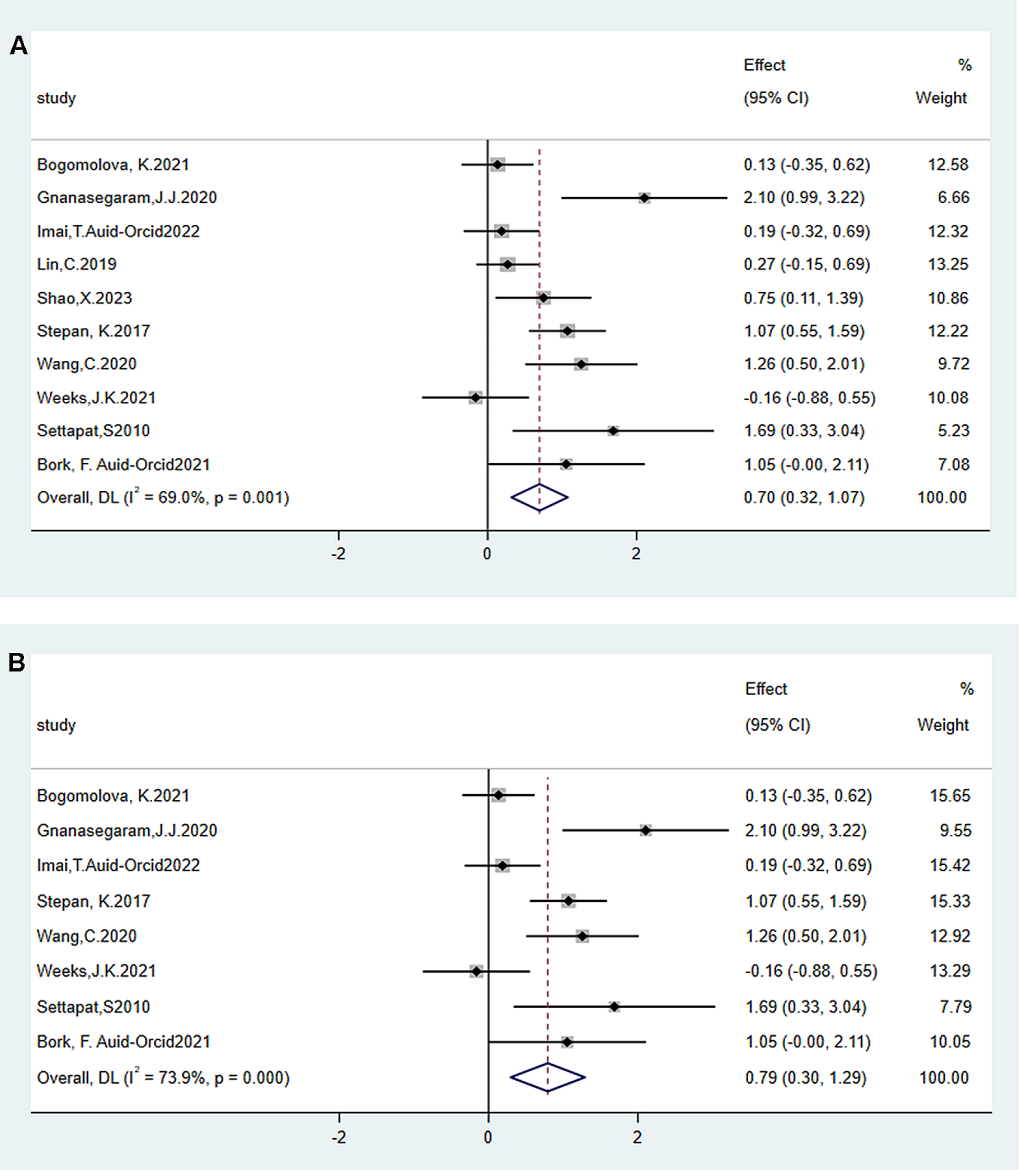
Comparison of the experimental and control groups for enjoyment outcome

We included six documents on time consumption [20, 24, 25, 29, 46, 51] and four documents in the forest map of enjoyment value [32, 40, 45, 48]. The results showed no statistical difference between the 3D group and the traditional group (SMD = -0.55, 95% CI = -1.23–0.14, *p* > 0.05, I^2^ = 86.5%, Supplementary Figure S4A). If the study from China is removed, the statistical significance of the results remains unchanged (Supplementary Figure S4B). However, the results of the happiness value forest map show that 3D technology makes participants feel happier (SMD = 3.04, 95% CI = 1.05–5.04, *p* < 0.05, I^2^ = 95.8%, Fig. 6A). If the study from China is deleted, the statistical significance of the results remains unchanged as well (SMD = 2.73, 95% CI = 0.32–5.15, *p* < 0.05, I^2^ = 96.5%, Fig. 6B).

### Publication bias

According to the results, the funnel chart is basically symmetrical, with a vertical line in the middle representing the combined OR value, and all studies are generally evenly distributed on both sides of the vertical line, showing an inverted funnel shape (**Figures ****S5****A-C**). This shows no obvious bias in grades, test time, or satisfaction. At the same time, the results of the Egger’s test for test time and test performance showed non-significant asymmetry (*p* > 0.05); therefore, no apparent application bias was observed in the present study. However, the Egger’s test of satisfaction showed application bias (*p* < 0.05), and when the Chinese studies were removed, no application bias was found *(p* > 0.05).

### Sensitivity analysis

**Fig. 7:**
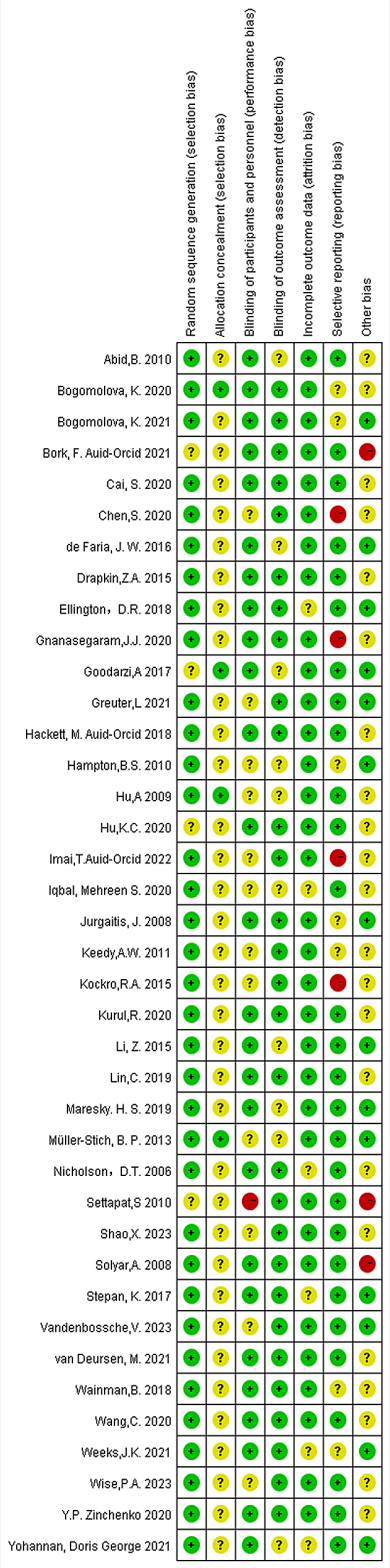
**(A)** Sensitivity analysis of the test results of the experimental and control groups was performed by meta-analysis. **(B)** The sensitivity analysis of the satisfaction of the experimental and control groups was conducted by meta-analysis

Due to the significant heterogeneity (I^2^ > 75%), we created a sensitivity analysis chart to verify the reliability of the results. We found that when any research was removed from the model, the significant influence of 3D visualization on test scores, satisfaction, and test time remained unchanged (Fig. 7A and B, Figure S6). Therefore, this result shows that the survey’s inspection results were reasonable.

### Regressive analysis

To confirm the influence of various factors, we made a meta-regression analysis on the influence of four potential factors: learners, countries, courses, and time (Table 1). We grouped medical students versus other learners, China versus other countries, and neuroanatomy versus other anatomy. The results show that the P-value of the country is less than 0.05, which indicates that the national factors have a significant influence on the results, while the other factors have none.

**Table 1:** Meta-regression analysis for different subgroups

Factors	Coefficient	Standard error	P	95% CI	
Participant	0.46	0.61	0.46	-0.81	1.74
Year	-0.7	0.45	0.14	-1.64	0.25
Course	-0.63	0.5	0.22	-1.68	0.42
Country	-1.47	0.5	0.01	-2.51	-0.44

## Discussion

In the recent 10 years, 3D visualization technologies such as VR, augmented reality, and mixed reality have become increasingly popular [5].This meta-analysis included 39 studies; interestingly, for countries with the highest number of studies, these studies are directly related to their economy and technology. For example, the United States, the largest economy in the Americas, is also the region with the highest number of studies. China, the country with the largest economy in Asia, has the highest number of Asian studies. Germany, the leading country in the European economy, has the highest number of studies in Europe. Generally, the risk of bias in most studies is due to a lack of data or unclear descriptions as well as to other descriptions [57]. In all 39 studies, the subjects were divided into random control groups, and the heterogeneity may have been caused by differences in teaching quality and test difficulty in different schools.

Most medical students learn about anatomy using traditional textbooks. Autopsy is a special teaching method that has many advantages but also limitations [58]. Therefore, if 3D technology is widely used in this subject, it can improve students’ understanding of three-dimensional graphics [59]. In addition, 3D technology has potential practicability not only in education/training but also in operation planning and intraoperative guidance [60].From a learning perspective, 3D visualization technology can stimulate students to explore their own understanding [61]. This is beneficial to their clinical diagnosis and treatment after study. However, whether 3D technology can improve participants’ test scores varies depending on the research findings. There are many conclusions from previous meta-analyses. Some have shown that 3D visualization technology can improve learner performance [57], while others have shown that 3D visualization is a more effective method for acquiring anatomical knowledge than traditional methods [62].Others also show that 3D visualization as a learning tool has potential beneficial effects on learning [63].However, we disagree with this viewpoint. Thus, we included a greater number of studies in our research compared to them. Similarly, a literature summary including all regions found that the 3D group performed better in the test than the control group. However, after excluding studies from China, the results changed. They may have a regional bias, with higher positivity rates in the study from China, and we cannot rule out the possibility that the authors might have had a preference for positive results to publish the study. Therefore, whether 3D visualization can improve test scores is a question that carries substantial weight, and we are more inclined not to answer it. Excitingly, regarding the cost of answering questions, satisfaction, enjoyment, excluding and not excluding literature from China, after merging the data, we found that the results were stable. Therefore, the credibility of our findings is high.

The advantage of this research lies mainly in the retrieval and extraction of the literature, and we developed our own literature screening process. Compared with most document screening processes, our two-step document screening method is more accurate and comprehensive; this significantly improves the efficiency of document screening. In addition, compared with most related studies, we included more documents. Although these technologies provide interesting, new pedagogical possibilities, they have some limitations [9], including restrictions on the conditions of use, such as the high cost of 3D visualization technology, which makes it difficult to popularize.Undeniably, the limitations of our meta-analysis also include that some related papers may be omitted. Furthermore, learners’ different understandings of space affected the experimental results. However, it takes considerable skill and practice to develop the ability to visualize in three dimensions and insufficient ability to visualize frequently expressed by the students [64]. Due to the small number of participants in some studies, the experimental data may have subjective influence.

## Conclusion

Medical students and residents who use 3D visualization technology to learn about human anatomy do not improve their test scores. Regional factors (the countries to which the 39 included studies belong) have a significant impact on the test results; the literature from China is more likely to have positive results, whereas other factors, such as learners, courses, and time, have no significant impact. 3D technology cannot shorten the time required for participants to answer questions; however, it can improve the participants’ satisfaction and enjoyment. Overall, 3D visualization technology is a promising teaching-aid technology.

### Electronic supplementary material

Below is the link to the electronic supplementary material.

Supplementary Material 1

Supplementary Material 2

Supplementary Material 3

Supplementary Material 4

Supplementary Material 5

Supplementary Material 6

Supplementary Material 7

Supplementary Material 8

## Data Availability

All data and materials are available as supplementary materials.
