# Supplementary figures and images for: 3D visualization technology for Learning human anatomy among medical students and residents: a meta- and regression analysis
Source: BMC Med Educ. 2024 Apr 26;24:461. doi: 10.1186/s12909-024-05403-4 (PMC11055294; doi:10.1186/s12909-024-05403-4)

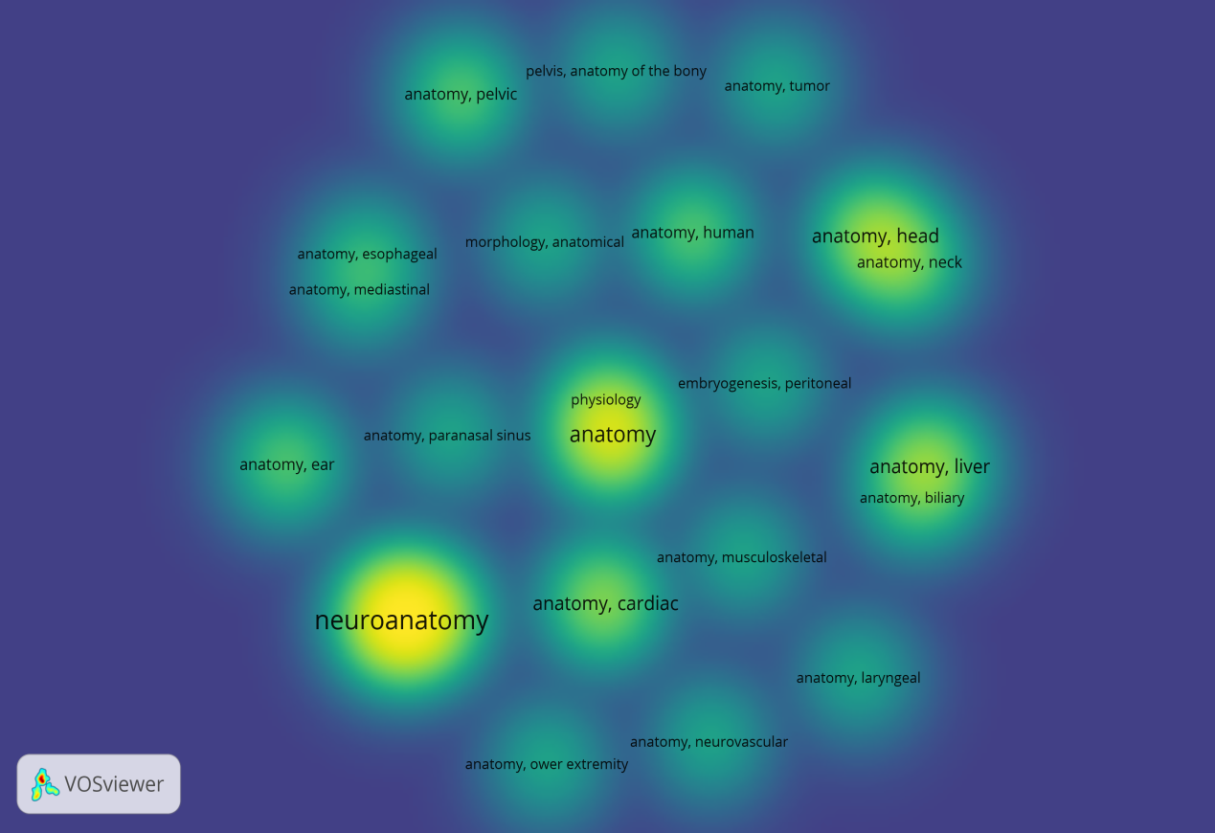

Supplement: Supplementary file 3 — Supplementary Material 3 [file 12909_2024_5403_MOESM3_ESM.tif]

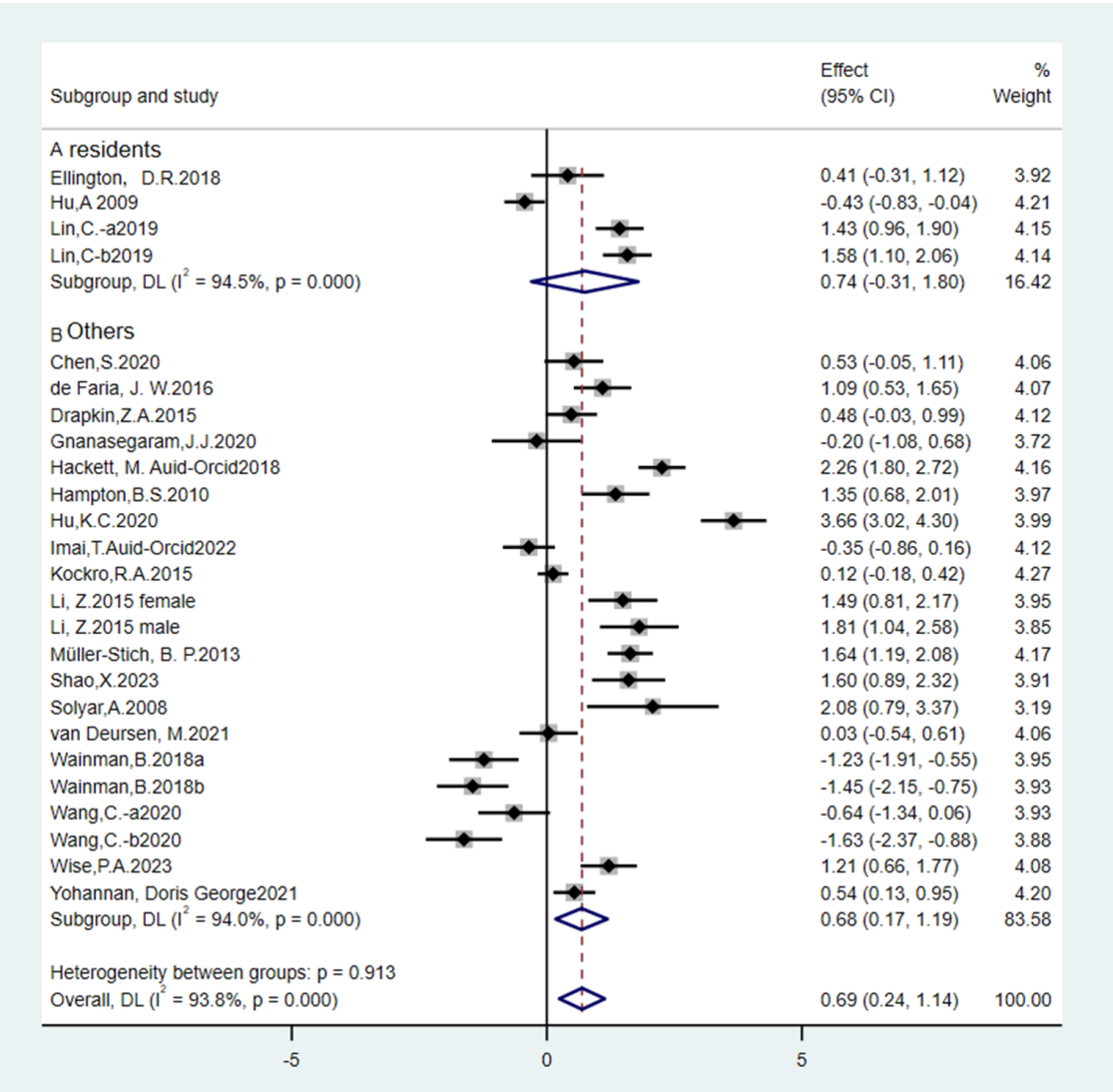

Supplement: Supplementary file 4 — Supplementary Material 4 [file 12909_2024_5403_MOESM4_ESM.tif]

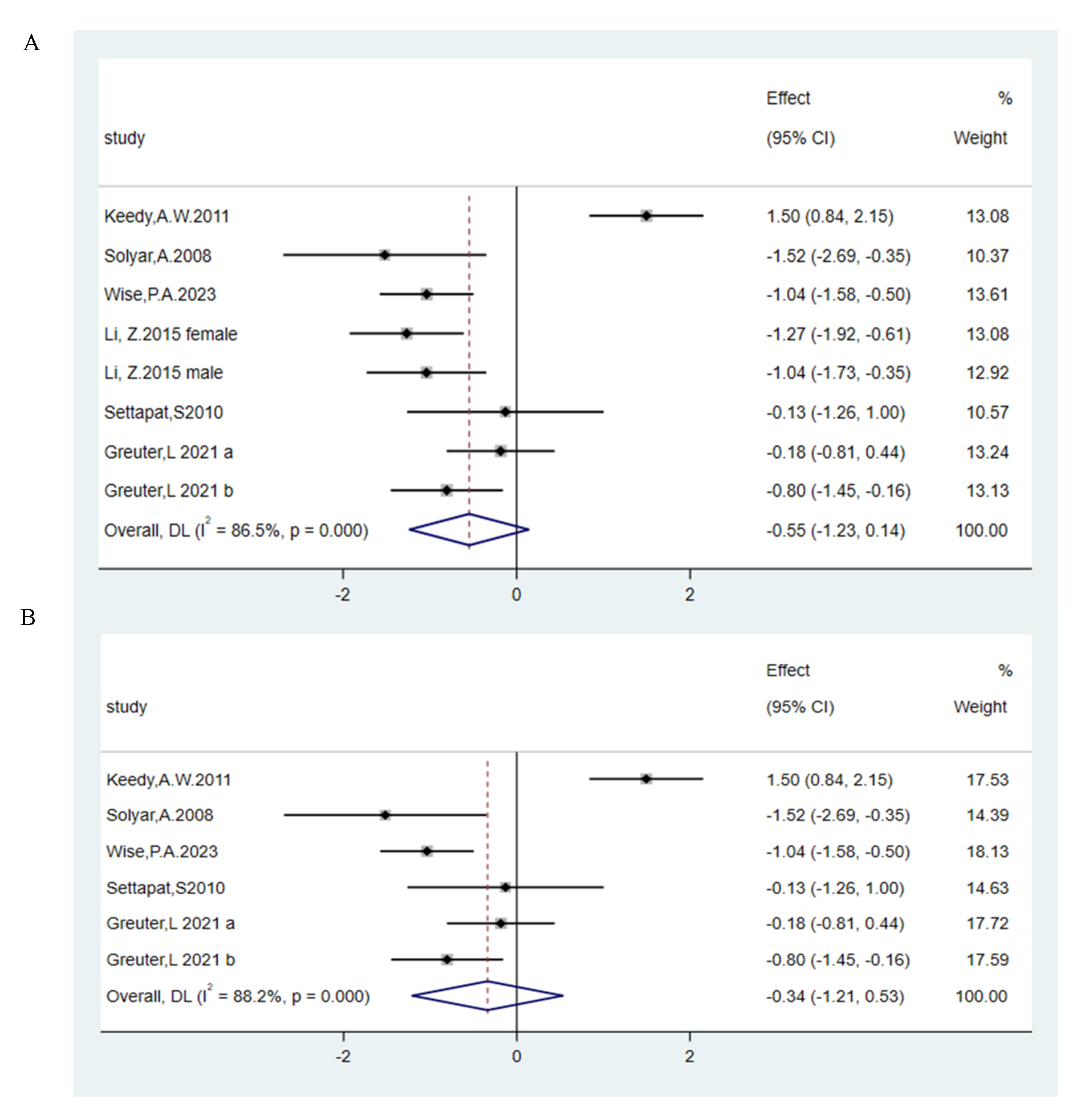

Supplement: Supplementary file 6 — Supplementary Material 6 [file 12909_2024_5403_MOESM6_ESM.tif]

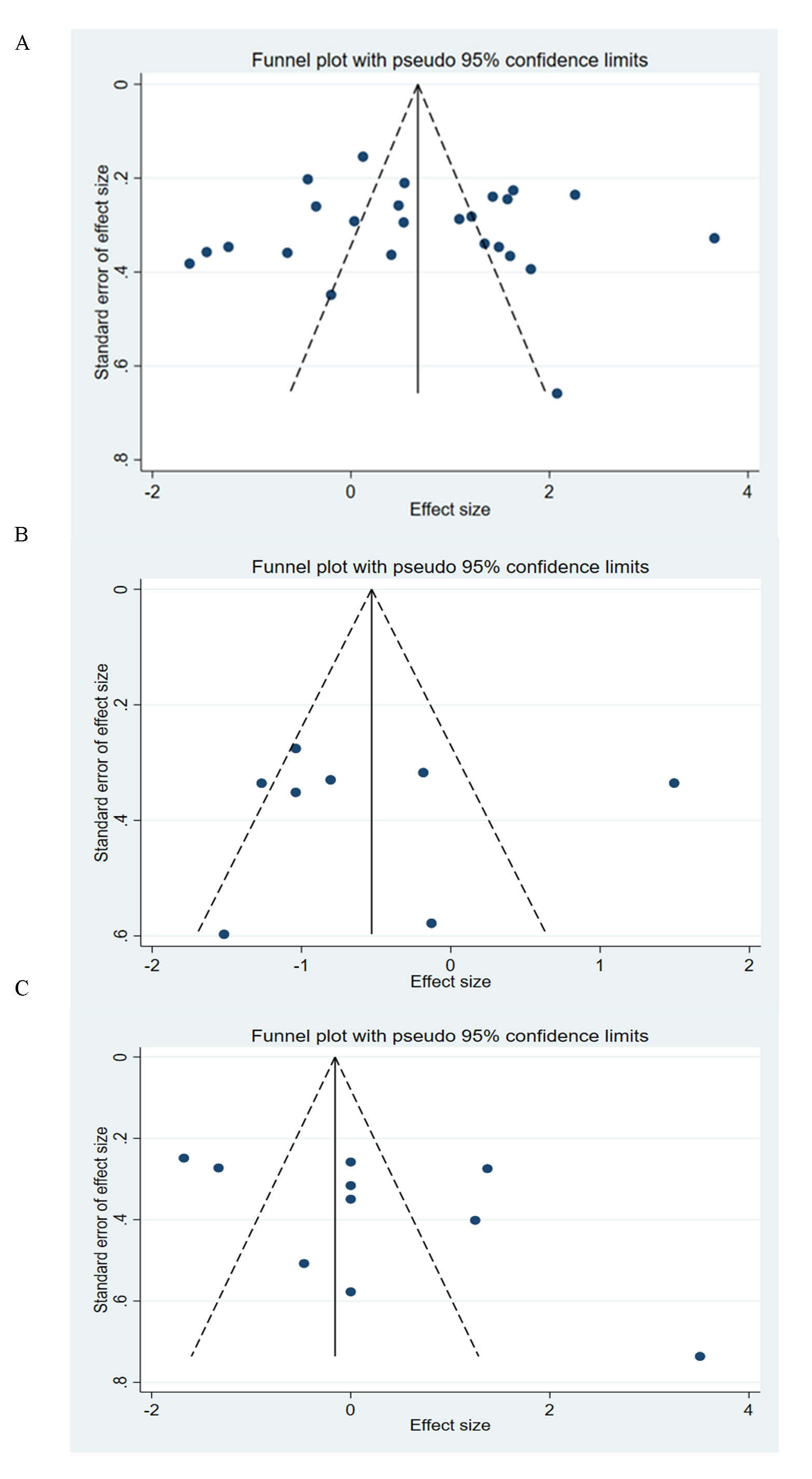

Supplement: Supplementary file 7 — Supplementary Material 7 [file 12909_2024_5403_MOESM7_ESM.tif]

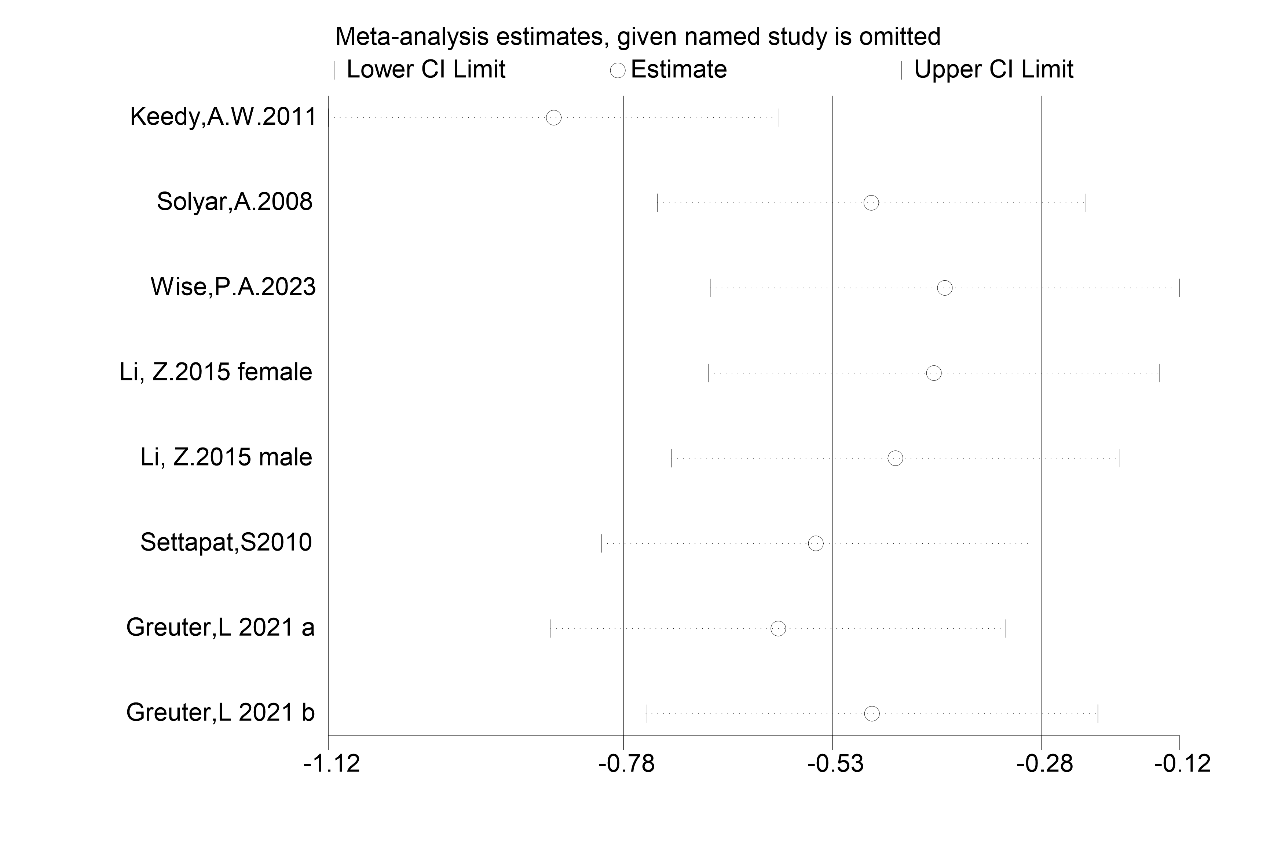

Supplement: Supplementary file 8 — Supplementary Material 8 [file 12909_2024_5403_MOESM8_ESM.tif]
